# Budget Impact Analysis of Stepping Down Patients from Long-Term Inappropriate Proton Pump Inhibitor Use to Episodic Alginate Treatment: NHS England Perspective

**DOI:** 10.36469/001c.144254

**Published:** 2025-10-30

**Authors:** Joshua Wray, Patricia Aluko, Yuvraj Sharma, Erzsebeth Horvath, Manisha Panchal, Ines Guerra, Richard Stevens, Cathal Coyle, Kate Plehhova

**Affiliations:** 1 Reckitt, Hull, UK; 2 IQVIA, London, UK; 3 IQVIA, Bangalore, India; 4 Primary Care Society for Gastroenterology, Oxford, UK

**Keywords:** budget impact model, proton pump inhibitors, alginate therapy, gastroesophageal reflux disease, NHS cost savings

## Abstract

**Background:**

Long-term inappropriate use of proton pump inhibitors (PPIs) for the treatment of gastroesophageal reflux disease (GERD) and dyspepsia is a common issue that can lead to unnecessary healthcare costs and potential adverse effects. Alternative treatments, such as episodic alginate therapy, may offer a more cost-effective and clinically appropriate approach.

**Objective:**

To investigate the financial impact of transitioning patients from long-term inappropriate PPI use to episodic alginate treatment for GERD and dyspepsia, from the perspective of the National Health Service (NHS) England.

**Methods:**

A budget impact model was used to compare costs over a 5-year period for adult patients using long-term inappropriate PPIs, with and without alginate treatment. By the fifth year, 20% of patients were assumed to have switched to alginate treatment. In this model, the base case analysis included only drug costs, while a scenario analysis also considered adverse effect costs.

**Results:**

Over the 5-year period, net savings of £11.5 million were observed in drug acquisition costs when 20% of patients (4.8 million) successfully transitioned to alginate treatment. When adverse effect costs were included in the scenario analysis, net savings increased to £16.6 million due to a slight reduction in the number of adverse effects. One-way sensitivity analysis confirmed the robustness of these results.

**Conclusions:**

Transitioning patients from long-term PPI use to episodic alginate treatment is beneficial for patients, potentially reducing adverse effects, and can lead to significant budgetary cost savings, which can be reallocated.

## INTRODUCTION

Gastroesophageal reflux disease (GERD) is characterized by leakage of caustic stomach contents, such as acid, pepsin, and bile, into the esophagus, causing symptoms such as heartburn and an unpleasant sour taste at the back of the mouth.^1^ Dyspepsia, another common upper gastrointestinal disorder,^2^ describes a range of symptoms such as upper abdominal pain or discomfort, retrosternal pain, gastric reflux, nausea, or vomiting.^3^ The prevalence of GERD is estimated to be 9% to 26% in Europe, with an incidence of 5 per 1000 person-years in the United Kingdom (UK).^4^ Prevalence of dyspepsia may vary from 12% to 41% in the UK according to the definition used for dyspepsia.^5^ These two conditions often show symptomatic overlap, with about half of the patients with dyspepsia having symptoms associated with GERD.^2,6^

The National Institute for Health and Care Excellence (NICE) guidelines in England recommend lifestyle changes prior to pharmacological interventions for patients with GERD and dyspepsia.^3,7^ In cases where pharmacotherapy is needed, 4 to 8 weeks treatment with a proton pump inhibitor (PPI) is recommended.^3^ Long-term PPI maintenance therapy is suggested only for patients with refractory or recurrent symptoms following initial management, complicated or severe esophagitis, Barrett’s esophagus, or when they require a PPI for gastroprotection while taking nonsteroidal anti-inflammatory drugs (NSAIDs) for a prolonged period. Continuous PPI treatment is also considered for patients with previous bleeding peptic ulcer disease who are *Helicobacter pylori*–positive after at least 2 attempts at eradication.^3^ However, it is strongly recommended to annually review the condition of these patients and encourage them to step down to the lowest effective dose or stop PPI treatment if appropriate.^3^ Despite these guidelines, inappropriate use of PPIs has often been observed in clinical practice.^8–10^ Physicians tend to prescribe PPIs as first-line treatment for acid-related conditions, for prolonged durations, or for inappropriate indications.^11–14^ Results from a cross-sectional study in the UK showed that nearly 38% of patients use PPIs for over a year.^15^ Prolonged PPI use has been associated with increased risk of enteric infections such as *Clostridium difficile* and community-acquired pneumonia, mainly because PPIs reduce gastric acidity, which makes the intestine easily accessible for microorganisms or can lead to gut microbiota dysbiosis.^16^ Observational studies have also shown association of long-term and inappropriate use of PPIs with kidney diseases and risk of bone fractures; however, the mechanism of these associations could not be clearly defined.^16^

In line with these potential adverse events, NICE guidelines recommend that patients who do not require clinically justified long-term PPI therapy (eg, those without Barrett’s esophagus, severe erosive esophagitis, or those not requiring gastroprotection for long-term NSAID use) should be reviewed annually and, where appropriate, gradually stepped down by encouraging the use of the lowest effective dose, considering “as needed” usage when appropriate, and returning to self-treatment with antacids and/or alginates.^3^ However, one of the biggest hurdles of discontinuation of PPIs is rebound acid hypersecretion.^17^ Currently, clinical guidelines lack a clear management strategy for rebound symptoms, thus creating a barrier for patients to discontinue PPI. Since duration of PPI use is a predictive factor for symptom recurrence after step-down, long-term use makes deprescription even more challenging.^18,19^

One approach to control rebound symptoms in patients stepping off PPIs could involve alginates, which provide an alternative mode of action by reducing the flow of acidic refluxate and creating a mechanical barrier displacing the postprandial acid pocket.^20^ They can be used as monotherapy or in combination with PPIs^14^ and have shown noninferior clinical efficacy compared with PPIs in patients with GERD.^20,21^ Alginates have been shown to provide significant relief from reflux symptoms in patients with GERD who were on PPIs in randomized trials.^22,23^ Moreover, regular alginate use has been shown to be effective in managing symptoms of rebound acid hypersecretion 1 week after cessation of long-term PPIs.^24^ A real-world, prospective study in the UK also showed that alginates, along with educational material on self-care, resulted in 75% of individuals successfully stepping down or stepping off PPI over a year.^25^ In the UK, over £116 million were spent annually on PPIs in 2014.^26^ Hence, stepping patients off PPIs and encouraging alginate use as short-term rescue therapy, where alginates can be utilized as immediate symptom relief supporting the patient during PPI dose reduction (episodic therapy), may help reduce the risks and additional economic burden associated with long-term inappropriate PPI use.

Therefore, the aim of this study was to investigate the impact of actively stepping patients down/off long-term PPIs to episodic alginate treatment in the management of GERD and dyspepsia, from the perspective of the National Health Service (NHS) England using a budget impact model (BIM). In this study, ‘episodic’ alginate is defined as the use of alginates on an “as-needed basis” during symptomatic reflux or dyspepsia episodes, rather than daily maintenance therapy.

## METHODS

### Model Structure

A 5-year time horizon was considered appropriate from an NHS perspective for budget planning as recommended by the International Society for Pharmacoeconomics and Outcomes Research (ISPOR) guidelines.^27^

**Figure 1.**
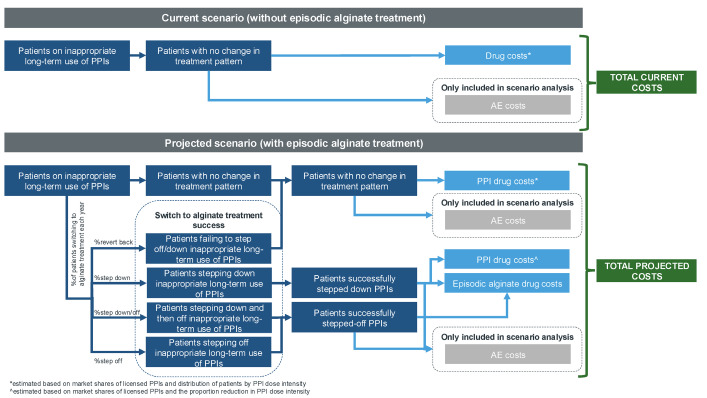
Budget Impact Model Scenarios Abbreviations: AE, adverse event; PPI, proton pump inhibitor.

The model estimated the impact of actively stepping down/off patients from long-term PPIs to episodic alginate treatment (ie, alginate is used as needed during symptomatic episodes rather than continuously) in patients with GERD and dyspepsia in England. The model considered two budget scenarios (**Figure 1**): (1) without episodic alginate treatment and (2) with episodic alginate treatment.

The target population was stratified into subgroups based on age (18-39, 40-59, ≥60 years) and duration of PPI use (3-6 months, 6-12 months, 1-5 years, >5 years) (**Supplementary Figure S1**). These subgroups were used to estimate the proportion of patients considered to be on inappropriate long-term PPIs and eligible for episodic alginate therapy. The current scenario derived the total current costs (drug costs only) from patients on inappropriate long-term PPIs with no change to their treatment pattern. The projected scenario stratified patients into patients with no change in therapy, patients stepping down/off PPIs, and those successfully switching to alginate treatment including the probability of reverting back to prior PPI use. Costs were estimated using market shares of licensed PPIs, distribution of patients by PPI dose intensity, and the proportion of patients moving between treatment patterns each year.

### Model Inputs

**Patient population**: The patient population included adult patients (≥18 years) identified as having inappropriate long-term PPI usage. Inappropriate use of PPI was defined as using PPIs for indications without a disease code or using them for durations longer than the recommended 3 months without a corresponding indication for long-term use (eg, Barrett’s esophagus or erosive esophagitis), or with a short-term indication code (eg, GERD, dyspepsia).^3,28^ A full list of appropriate long-term indication for PPI use is provided in **Supplementary Table S1**.^3,29^

The target population eligible for treatment with alginate in the base year (year 1) was estimated using a cascade approach (**Table 1**). Newly eligible patients were added each year of the time horizon until year 5. A population growth rate of 0.80% per year was applied to estimate the total eligible patient population each year.^30^

**Table 1.** Population and Market Shares Inputs

**Parameter**	**Value**	**Source/Comments**
Estimation of eligible population		
Total population in England (year 1)^a^	57 699 579	Office for National Statistics^31^
Adults (18-39 years)	28.94%	
Adults (40-59 years)	25.89%	
Adults (≥60 years)	24.73%	
Patients receiving PPI (18-39 years)	6.15%	Abrahami et al^15^
Patients receiving PPI (40-59 years)	16.51%	
Patients receiving PPI (≥60 years)	32.15%	
Patients considered inappropriate long-term PPI	95.63%	Plahhova et al^28^
Patients eligible for treatment with alginate treatment	100.00%	Assumption based on Gaviscon® prescribing information^32,33^
Patients eligible for treatment with alginate treatment by age		
18-39 years	7.33%	Plahhova et al^28^
40-59 years	28.15%	
≥60 years	64.51%	
Patients eligible for treatment with alginate treatment by duration of PPI use^b^		
3-6 months	0.42%	Plahhova et al^28^
6-12 months	1.16%	
1-5 years	12.09%	
>5 years	86.33%	
PPI dose intensity (proportion of patients by initial dose)		
Low dose	17.79%	Plahhova et al^28^
Full dose	67.49%	
Double dose	14.71%	
Step-down/off success (proportion of patients stepping down/off from inappropriate PPI long-term use at 12 months)		
Step down	34.77%	Coyle et al^25^
Step down then off^c^	4.99%	
Step off only	35.30%	
Revert	24.93%	
Step-down/off success - PPI dose reduction ratio		
Step down	0.25	Assumption
Step down then off	0.25	
Step off only	0.00	
Revert	1.00	
Market shares of PPIs without alginate treatment^d^		
Esomeprazole	3.59%	NHS Business Services Authority^34^ Assumed same market shares for years 1-5
Lansoprazole	45.06%	
Omeprazole	48.59%	
Pantoprazole	2.36%	
Rabeprazole	0.39%	

Abbreviations: NHS, National Health Service; PPI, proton pump inhibitor.

^a^Population growth per year (0.80%).^30^

^b^Patients with duration of treatment (time since first PPI issue).

^c^ Assumes 75% reduction in dose for first 6 months then complete step-off.

^d^For scenario with alginate treatment, progressive increase in market share of alginate from 10% in year 1 to 20% in year 5 was assumed with a proportionate decrease in PPI market shares each year.

The target population was further segregated into risk groups based on age (18-39 years, 40-59 years, and ≥60 years) and duration of PPI use (3-6 months, 6-12 months, 1-5 years, and >5 years) (**Table 1**), following the distribution observed in a previous study exploring inappropriate PPI prescribing patterns in England.^28^

**Intervention and comparators**: Episodic alginate treatment, where patients used alginate as needed during symptomatic episodes, was used as the intervention, while licensed PPIs (esomeprazole, lansoprazole, omeprazole, pantoprazole, and rabeprazole) were used as comparators.

**Treatment shares**: A gradual stepping-off approach was employed to account for patients switching to alginate treatment. It was assumed that 20% of patients would be stepped off long-term PPI use to alginate treatment by year 5. The existing PPI therapies were assumed to be displaced at a rate proportional to their current market share.

**Step-down/off success**: For patients switching to alginate treatment each year, a step-down/off success rate was applied to account for a successful/unsuccessful switch (**Table 1**). The patients switching to alginate treatment each year were split into four categories:

1. **Step down**: Patients who stepped down to a lower dose of PPI in combination with alginate treatment
2. **Step down and then off**: Patients who stepped down to a lower dose of PPI in combination with alginate treatment initially, and then stopped PPI use (assumed to be 6 months after alginate treatment initiation)
3. **Step off**: Patients who completely stepped down from day 1 and switched to alginate treatment
4. **Revert**: Patients who initiated alginate treatment in combination with their original PPI dose, but stopped alginate treatment after 12 months and continued on their original PPI dose

A PPI dose reduction ratio was applied to each of the four categories of patients switching to alginate treatment each year (**Table 1**), which was maintained through the remaining model time horizon. These success rates are based on a nurse-led deprescribing program^25^; the implementation into routine NHS practice could be met with some challenges.

**Costs**: Since this BIM is based on a smaller time horizon, we focused on the immediate costs of treatment (ie, drug costs). We excluded the cost of adverse effects (AEs) from the base case analysis, as they will be realized only at longer period. Also, drug acquisition costs would be beneficial in revealing the savings due to the inclusion of episodic alginate treatment compared with daily PPIs. These short-term savings could lead to potential savings for the healthcare system over longer periods. The long-term cost savings, including the cost of AEs, would be better explored through a cost-effectiveness analysis. The base case was therefore revised to include both drug acquisition costs and AE costs, with a sensitivity analysis presented for drug costs only.

The drug acquisition costs, which were obtained from the British National Formulary 2024, reflect the lowest unit cost of available dispensing options (**Supplementary Table S2**). All costs were reported in British pounds (2024). The PPI dose was based on the distribution of patients by PPI dose intensity (**Table 1**), as observed in a previous study exploring the prescribing practices for PPIs among primary care physicians in England.^28^

To estimate the annual dose of alginate treatment, data from Coyle et al^25^ were utilized. This was a large scale, real-world, nurse-led, PPI deprescribing study conducted in the UK enrolling 6249 eligible patients and using the Dyspepsia Therapy Review and Education Programme (DTREP). During the 12 months of the DTREP, PPI prescriptions decreased from 89 915 to 45 880 while alginate prescriptions increased from 2405 to 6670. An average of 1.7 bottles (500 ml each) of Gaviscon Advance® were used by patients who stepped down and/or off PPI use.^25^

**Adverse effects**: The AE rates in the model were estimated by converting the long-term risk of AEs into annual probabilities of an event, using rate-probability conversion and assigning it an event cost. Therefore, the base case analysis excluded the cost of AEs due to the short time horizon of the BIM, which is not optimal for capturing long-term AEs; they are only presented as a scenario. Although AE costs are important, their inclusion would be more appropriate in a cost-effectiveness model, so further research utilizing the model is recommended. The rates of AEs were retrieved from national sources or from published literature (**Supplementary Table S3**).

The relative risk of developing AEs due to long-term PPI use was obtained from published sources and was segregated based on the population age groups and duration of PPI use (**Table 1**). For alginate, the population-based incidence rates of the AEs were assumed while the relative risk values of developing AEs through PPI use were applied for patients on PPIs. Based on alginate periodic safety update reports, it was assumed that alginate does not increase probability of long-term AEs. Moreover, the use of alginate was episodic. Unit costs associated with each AE were obtained either from the NHS reference costs^35^ (available for 2023 at the time of analysis) or from published sources. Costs from published sources were inflated to 2023 units using the Unit Costs of Health and Social Care 2023 Manual.^36^

### Model Outputs

The model results were presented as net budget impact and total costs with and without alginate treatment. Additionally, the number of patients treated with alginate treatment over the 5-year time horizon was reported.

**Scenario and sensitivity analysis**: Scenario analyses and one-way sensitivity analysis (OWSA) were performed to test the model robustness and identify model drivers. A scenario with AE costs included was explored, in addition to the drug acquisition costs, to assess the impact of potential savings due to reduction in AEs. Additional scenario analyses were conducted to test assumptions around alginate treatment market shares and PPI step-down/off success rates.

OWSA was performed for cost and population input parameters to ascertain parameter uncertainty and their impact on the total budget impact. The values of these input parameters were varied within 20% of the base case values. This deterministic sensitivity analysis is in agreement with the NICE guidelines that suggest the use of deterministic sensitivity analysis for identifying parameters to which the decision is most sensitive when there are influential but highly uncertain parameters.^37^

## RESULTS

The model estimated that approximately 4.8 million patients would successfully step down/off to episodic alginate treatment from PPIs over a time horizon of 5 years (**Supplementary Table S4**). The proportion of patients successfully stepping down/off to alginate treatment would gradually increase from 0.77 million in year 1 to 1.25 million by year 5 accompanied by a reduction in patients using PPIs. This represents approximately 20% of the eligible population and corresponds to a 3.5% reduction in overall budget impact.

### Base Case Analysis

The base case analysis showed that total drug acquisition cost for the eligible population over a 5-year time horizon was £494 million without alginate. The use of alginate treatment led to reduction in the total cost to £482 million (**Table 2**). Over the 5-year time horizon, the drug acquisition cost for PPIs were £440 million and the acquisition cost for alginate was £42 million. The net savings increased gradually with each year of stepping down/off PPI to alginate treatment (£1.8 million in year 1 to £3.0 million in year 5). Overall, stepping down/off 20% of the patients on PPI to alginate treatment resulted in a net savings of £11.5 million for the eligible population (**Table 2**).

**Table 2.** Results of the Base Case Analysis for the Entire Eligible Population

**Parameter**	**Values**					
**Year 1**	**Year 2**	**Year 3**	**Year 4**	**Year 5**	**Total**	
Total eligible population, n	7 727 893	7 789 703	7 852 007	7 914 809	7 978 114	39 262 527
Patients treated with episodic alginate therapy, n	772 789	781 041	935 035	1 091 442	1 250 292	4 830 600
Drug acquisition costs without alginate treatment, £						
PPI	97 207 129	97 984 617	98 768 323	99 558 298	100 354 591	493 872 957
Drug acquisition costs with alginate treatment, £						
Alginate	6 726 358	6 798 182	8 138 545	9 499 915	10 882 541	42 045 541
PPI	88 692 957	89 318 864	88 390 176	87 440 918	86 470 821	440 313 737
Total drug acquisition costs, £	95 419 316	96 117 046	96 528 721	96 940 833	97 353 362	482 359 278
Net budget impact, £	−1 787 813	−1 867 570	−2 239 602	−2 617 465	−3 001 229	−11 513 679

Abbreviation: PPI, proton pump inhibitor.

### Scenario Analysis

In the scenario where costs of AEs were included in the analysis, the total number of AEs was found to be marginally lower in the population with alginate treatment than those without alginate treatment (**Table 3**). This scenario analysis showed that the total cost over a 5-year time horizon was reduced if alginate treatment was included (£818 million) compared with PPI use only (£834 million) (**Table 3**). Cost-breakdown by category showed that both drug acquisition cost (£482 million vs £494 million) and cost associated with AEs (£336 million vs £341 million) were lower in the scenario where alginate was introduced. Overall, switching 20% of the patients to alginate treatment resulted in a net savings of £16.7 million compared with the scenario with only PPIs over a 5-year period (**Table 3**).

**Table 3.** Results of Scenario Analysis Including Adverse Effect Costs

**Parameter**	**Costs (£)**					
**Year 1**	**Year 2**	**Year 3**	**Year 4**	**Year 5**	**Total**	
Costs without alginate treatment						
Drug acquisition: PPI	97 207 129	97 984 617	98 768 323	99 558 298	100 354 591	493 872 957
AE: PPI	67 108 694	67 645 447	68 186 493	68 731 866	69 281 602	340 954 102
Total cost	164 315 823	165 630 064	166 954 816	168 290 164	169 636 192	834 827 060
Drug acquisition costs with alginate treatment						
Alginate	6 726 358	6 798 182	8 138 545	9 499 915	10 882 541	42 045 541
PPIs	88 692 957	89 318 864	88 390 176	87 440 918	86 470 821	440 313 737
Total	95 419 316	96 117 046	96 528 721	96 940 833	97 353 362	482 359 278
AE costs						
Alginate	5 851 918	5 535 067	6 695 206	7 873 549	9 070 312	35 026 052
PPI	60 397 825	61 297 936	60 508 556	59 702 628	58 879 937	300 786 883
Total	66 249 743	66 833 003	67 203 762	67 576 177	67 950 250	335 812 935
Total cost	161 669 058	162 950 049	163 732 484	164 517 010	165 303 612	818 172 213
Cost difference (with alginate vs without alginate)						
Drug acquisition	−1 787 813	−1 867 570	−2 239 602	−2 617 465	−3 001 229	−11 513 679
AE	−858 952	−812 444	−982 731	−1 155 689	−1 331 352	−5 141 168
Net budget impact	−2 646 765	−2 680 014	−3 222 332	−3 773 154	−4 332 581	−16 654 847

Abbreviations: AE, adverse effects; PPI, proton pump inhibitor.

Additional scenario analyses testing assumptions around alginate treatment market shares showed a linear increase in potential cost savings with increase in alginate treatment market shares (**Table 4**). The net savings increased to £23.4 million and £44.9 million compared with the scenario with only PPIs for scenarios, with 50% and 100% alginate treatment market shares over a 5-year period, respectively. For scenarios testing PPI step-down/off success rates, the net savings ranged from £4.3 million, when all patients were assumed to step-down only, to £22.9 million, when all patients were assumed to completely step off PPIs.

**Table 4.** Results of Additional Scenario Analysis, Excluding Adverse Effect Costs

**Scenario**	**Costs (£)**					
**Year 1**	**Year 2**	**Year 3**	**Year 4**	**Year 5**	**Total**	
**Scenario analysis 1**: Alginate treatment market share 50% by year 5						
Costs without alginate treatment	97 207 129	97 984 617	98 768 323	99 558 298	100 354 591	493 872 957
Costs with alginate treatment	95 419 316	94 765 462	94 095 044	93 407 840	92 703 624	470 391 287
Net budget impact	−1 787 813	−3 219 155	−4 673 279	−6 150 457	−7 650 967	−23 481 671
**Scenario analysis 2**: Alginate treatment market share 100% by year 5						
Costs without alginate treatment	97 207 129	97 984 617	98 768 323	99 558 298	100 354 591	493 872 957
Costs with alginate treatment	93 631 502	91 546 307	89 421 766	87 257 383	85 052 657	446 909 616
Net budget impact	−3 575 627	−6 438 310	−9 346 557	−12 300 915	−15 301 934	−46 963 342
**Scenario analysis 3**: Step-down/off success with 50% step down, 50% step off						
Costs without alginate treatment	97 207 129	97 984 617	98 768 323	99 558 298	100 354 591	493 872 957
Costs with alginate treatment	95 427 863	95 742 746	96 056 561	96 369 271	96 680 839	480 277 281
Net budget impact	−1 779 265	−2 241 871	−2 711 762	−3 189 027	−3 673 752	−13 595 677
**Scenario analysis 4**: Step-down/off success with 100% step off						
Costs without alginate treatment	97 207 129	97 984 617	98 768 323	99 558 298	100 354 591	493 872 957
Costs with alginate treatment	94 212 774	94 211 737	94 204 655	94 191 433	94 171 974	470 992 573
Net budget impact	−2 994 355	−3 772 880	−4 563 668	−5 366 864	−6 182 617	−22 880 384
**Scenario analysis 5**: Step-down/off success with 100% step down						
Costs without alginate treatment	97 207 129	97 984 617	98 768 323	99 558 298	100 354 591	493 872 957
Costs with alginate treatment	96 642 953	97 273 756	97 908 467	98 547 109	99 189 703	489 561 988
Net budget impact	−564 176	−710 861	−859 856	−1 011 189	−1 164 887	−4 310 969

### Sensitivity Analysis

**Figure 2.**
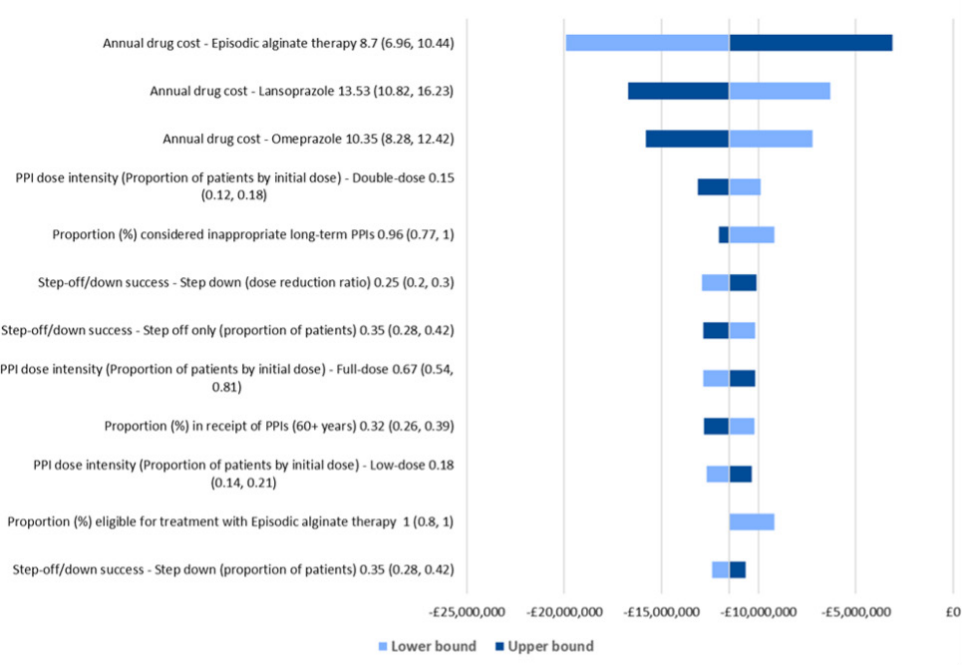
One-Way Sensitivity Analysis

When each of the parameters were varied by 20% in OWSA, the net budget continued to exhibit cost savings for patients stepping off PPIs to alginate treatment over 5 years. The analysis showed that the budget impact results were most sensitive to the annual drug costs of alginate, lansoprazole, and omeprazole and the proportion of individuals receiving double-dose PPIs initially (**Figure 2**). Overall, the net cost savings varied from £3 million to £20 million.

## DISCUSSION

Gradual stepping down/off long-term PPI use is recommended in the NICE guidelines for the treatment of GERD and dyspepsia. This analysis builds on previous studies exploring inappropriate PPI prescribing patterns in England^28^ and successful PPI step down approaches,^25^ to evaluate the budget impact of stepping down/off inappropriate long-term PPI use to episodic alginate treatment, allowing for as-needed use during symptomatic episodes, rather than continuously. Our results showed that the total cost of inappropriate long-term PPI use for the eligible population in England was £494 million. Stepping down/off only 20% of these patients to episodic alginate treatment reduced the total cost to £482 million. The cost saving over a 5-year period gradually increased from £1.8 million in year 1 to £3.0 million in year 5 in line with the increasing number of successful stepping down/off of patients eligible for alginate treatment each year (0.77 million in year 1 to 1.25 million in year 5). Net savings over the 5-year period for the eligible population was estimated as £11.5 million. The base case results were supported by scenario analysis (including cost of AEs), which showed that the cost savings were driven by both drug acquisition costs (70%) and the cost of managing AEs (30%). The net saving in this scenario was £16.6 million.

These results indicated that alginate treatment could help decrease the risks of AEs related to PPI use and reduce the overall cost under conservative switching assumption. The robustness of our base case results was validated by sensitivity analysis where the net budget continued to exhibit cost savings (£3 million–£20 million) for patients stepping down/off PPIs to alginate treatment. Alginate therapy would help in handing control back to the patients by providing relief from symptoms of GERD and dyspepsia as opposed to PPIs which often require long-term treatment and result in frequent treatment-related AEs.

To the best of our knowledge, this is the first study that evaluates the budget impact of stepping patients down/off inappropriate PPI use to alginate treatment. In addition, previously published studies have reported that alginate, used as short-term rescue therapy, can support patients in reducing or discontinuing long-term PPI use.^25^ In the study by Coyle et al, a net annual cost savings of nearly £30 000 was observed for all eligible patients who stepped off PPIs and were prescribed alginate treatment.^25^

It should be noted that this cost-saving included only drug costs while the costs of running the program or other medications used for dyspepsia were not considered.^25^ In the Netherlands, an intervention to discontinue inappropriate use of PPIs after cessation of NSAIDs or low-dose acetylsalicylic acid treatment had a per patient budget impact of €29 during the first year.^38^ However, the cost-utility analysis showed that this intervention was cost-saving (€79-€553) over the following 5 years with gain in QALYs.^38^ The cost savings were primarily driven by the reduced cost of AEs when PPIs were discontinued, as corroborated by our scenario analysis. Another patient-centered, nurse-led program in Scotland between 2008 and 2010 estimated the net savings when patients with long-term PPI use were encouraged to step down or step off with simultaneous prescription of alginate treatment.^39^

In this study, after 1 year of the program, annual costs of alginate prescription increased by £503 while those for PPI decreased by £3694 to £4521. The program led to a total annual cost saving of £3181 to £4008. In England, annual cost savings up to £7000 were observed with intervention to step down/off patients from long-term PPI use and encourage use of alginate for immediate relief of symptoms.^40^ It should be noted that the cost of running such deprescribing interventions and the cost to the primary care facilities could not be included in our model due to lack of appropriate data. Although not including these costs is a limitation, under the NHS medicine optimization strategy there is a current focus on deprescribing from a public management, clinical systems, and pharmacy perspective.^41,42^ Moreover, given that such interventions are one-time costs, it can be assumed that they would lead to cost savings in the long-term.

The results of this study must be interpreted in view of a few limitations inherent to real-world studies. The study employed a BIM with the aim of assessing the impact of deprescription of inappropriate long-term PPI use in terms of cost-savings for the NHS. Since a BIM is based on a smaller time horizon, only the immediate costs of treatment (ie, drug costs), were included for the base case analysis. A cost-effectiveness analysis would be more appropriate to capture long-term health benefits of reduction in PPI use at the patient level. The base case analysis in the study makes conservative assumptions, with only 20% of patients targeted to be stepped down/off to alginate treatment after 5 years and not including patients obtaining PPIs over the counter. Similarly, to estimate the eligible patient population, the rate of population growth was a conservative assumption, as PPI prescription growth is expected to exceed the rate of population growth. Additionally, the annual dose of alginate was estimated from the real-world study by Coyle et al.^25^ Although their study was based in the UK, they did not include other over-the-counter alginates or medications for dyspepsia. Therefore, the dose of alginate may be underestimated in our study.

Although it is worth noting that Gaviscon Advance has previously been found to be a cost-effective alternative compared to other most frequently prescribed alginate in the UK, Peptac.^43^ We also did not include the use of over-the-counter alginates and PPIs, as the study was conducted from the perspective of medicines prescribed by NHS England. Therefore, the actual proportion of population eligible for alginates and the use of PPIs could be much more than what was applied in our model.

We acknowledge that the study evaluates only one step down/off intervention and does not consider other available interventions such as histamine type-2 receptor antagonists (H2RAs) and antacids. While there is sufficient evidence showing similar efficacy of PPIs and alginates,^20,21^ reflecting the appropriateness of switching to alginates, there is lack of similarly robust evidence on comparative efficacy of PPIs and H2RAs/antacids.^44^

The primary limitation with many PPI deprescribing programs is their uncontrolled nature, with significant variance in their methodologies and many only reporting short-term interventions. Additionally, one step down/off method focused on as to not overcomplicate the analysis, especially as not everyone stepping down/off PPIs would be eligible to receive antacids.

Future analyses focusing on long-term risks associated with PPI overprescribing and comparative efficacy between the various step down/off interventions could provide additional insights into cost-effective strategies for deprescription of inappropriate long-term PPI use. It is worth noting that the implementation of such strategies across the NHS may face barriers such as prescriber inertia, lack of structured deprescribing support, and potentially underutilized pharmacist engagement, and success is likely to be more achievable when embedded in structured programs. However, medicine optimization is a key initiative in the NHS long-term plan, including services such as Structured Medication Reviews, and while PPIs are not a key focus of the service, polypharmacy is.^45^ In addition, primary care practices across the UK are increasingly employing clinical pharmacists who are optimally placed to help improve prescribing practices.^46^

This budget impact analysis would be beneficial from the NHS perspective in developing management strategies focusing on stepping patients down/off inappropriate long-term PPI use and switch to alternatives such as alginate treatment that is cost-saving while simultaneously reduces the risk of PPI-related AEs. Moreover, this strategy can support appropriate use and uptake of cost-effective medicines under the NHS medicines optimization policy by enabling cost savings in low-priority medicines such as PPIs.^41,42^ Eventually, such interventions can help physicians choose a patient-centric intervention for individuals who would otherwise be prescribed inappropriate PPI therapy.

## CONCLUSION

Stepping off patients from inappropriate long-term PPI use to episodic alginate treatment, where the symptomatic episodes are managed with alginate as needed, is not only in the best interest of the patient (reduced AEs) but can also lead to potential budgetary cost savings, which can be reallocated elsewhere. To maximize effectiveness, a structured deprescribing system may be considered.

### Disclosures

J.W., K.P., P.A., and C.C. are employees of Reckitt Benckiser. Y.S., M.P., and I.G. are employees of IQVIA and IQVIA received consulting fees from Reckitt. RS received consulting fees from Reckitt.

### Ethics Approval

Not applicable.

## Supplementary Material

Online Supplementary Material

## Data Availability

All data supporting the findings of this study are available within the article and its Supplementary Material.

## References

[1] NHS (2023). Heartburn and acid reflux.

[2] Quach D. T., Ha Q. V., Nguyen C. T. N.. (2022). Overlap of gastroesophageal reflux disease and functional dyspepsia and yield of esophagogastroduodenoscopy in patients clinically fulfilling the Rome IV criteria for functional dyspepsia. Front Med.

[3] NICE (2019). Gastro-esophageal reflux disease and dyspepsia in adults: investigation and management—Guidance.

[4] El-Serag H. B., Sweet S., Winchester C. C., Dent J. (2014). Update on the epidemiology of gastro-esophageal reflux disease: a systematic review. Gut.

[5] Kaji M., Fujiwara Y., Shiba M.. (2010). Prevalence of overlaps between GERD, FD and IBS and impact on health-related quality of life. J Gastroenterol Hepatol.

[6] (2019). National Institute for Health and Clinical Excellence Scope 1 Guideline Title.

[7] Mounsey A., Barzin Amir, Rietz A. (2020). Functional dyspepsia: evaluation and management. Pub Med.

[8] Heidelbaugh J. J., Kim A. H., Chang R., Walker P. C. (2012). Overutilization of proton-pump inhibitors: what the clinician needs to know. Ther Adv Gastroenterol.

[9] O'Mahony L., Yelverton E. (2019). Prescribing of proton pump inhibitors in an Irish general practice. Ir Med J.

[10] Pottegård A., Broe A., Hallas J., de Muckadell O. B. S., Lassen A. T., Lødrup A. B. (2016). Use of proton-pump inhibitors among adults: a Danish nationwide drug utilization study. Ther Adv Gastroenterol.

[11] Haastrup P., Paulsen M. S., Christensen R. D., Søndergaard J., Hansen J. M., Jarbøl D. E. (2016). Medical and non-medical predictors of initiating long-term use of proton pump inhibitors: a nationwide cohort study of first-time users during a 10-year period. Alimentary Pharmacol Ther.

[12] George C. J., Korc B., Ross J. S. (2008). Appropriate proton pump inhibitor use among older adults: a retrospective chart review. Am J Geriatr Pharmacother.

[13] Daniels B., Pearson S. A., Buckley N. A., Bruno C., Zoega H. (2020). Long-term use of proton-pump inhibitors: whole-of-population patterns in Australia 2013–2016. Ther Adv Gastroenterol.

[14] Plehhova K., Paquette N., Gould J., Coyle C. (2022). Understanding the patient PPI journey: results of a survey on PPI treatment initiation and patient experience. J Prim Care Community Health.

[15] Abrahami D., McDonald E. G., Schnitzer M., Azoulay L. (2020). Trends in acid suppressant drug prescriptions in primary care in the UK: a population-based cross-sectional study. BMJ Open.

[16] Castellana C., Pecere S., Furnari M.. (2021). Side effects of long-term use of proton pump inhibitors: practical considerations. Polish Arch Intern Med.

[17] Lødrup A. B., Reimer C., Bytzer P. (2013). Systematic review: symptoms of rebound acid hypersecretion following proton pump inhibitor treatment. Scand J Gastroenterol.

[18] Savarino E., Anastasiou F., Labenz J., Hungin A.P.S., Mendive J. (2022). Holistic management of symptomatic reflux: rising to the challenge of proton pump inhibitor overuse. Br J Gen Pract.

[19] Inadomi J. M., McIntyre L., Bernard L., Fendrick A. M. (2003). Step-down from multiple- to single-dose proton pump inhibitors (PPIs): a prospective study of patients with heartburn or acid regurgitation completely relieved with PPIs. Am J Gastroenterol.

[20] Leiman D. A., Riff B. P., Morgan S.. (2017). Alginate therapy is effective treatment for GERD symptoms: a systematic review and meta-analysis. Dis Esophagus.

[21] Pouchain D., Bigard M. A., Liard F., Childs M., Decaudin A., McVey D. (2012). Gaviscon® vs. omeprazole in symptomatic treatment of moderate gastroesophageal reflux. a direct comparative randomised trial. BMC Gastroenterol.

[22] Reimer C., Lødrup A. B., Smith G., Wilkinson J., Bytzer P. (2016). Randomised clinical trial: alginate (Gaviscon Advance) vs. placebo as add-on therapy in reflux patients with inadequate response to a once daily proton pump inhibitor. Alimentary Pharmacol Ther.

[23] Coyle C., Crawford G., Wilkinson J., Thomas S.J., Bytzer P. (2017). Randomised clinical trial: addition of alginate-antacid (Gaviscon Double Action) to proton pump inhibitor therapy in patients with breakthrough symptoms. Alimentary Pharmacol Ther.

[24] Vales A., Coyle C., Plehhova K., Hobson A., Woodland P. (2023). Randomised clinical trial: the use of alginates during preinvestigation proton pump inhibitor wash-out and their impact on compliance and symptom burden. BMJ Open Gastroenterol.

[25] Coyle C., Symonds R., Allan J.. (2019). Sustained proton pump inhibitor deprescribing among dyspeptic patients in general practice: a return to self-management through a programme of education and alginate rescue therapy. A prospective interventional study. BJGP Open.

[26] PrescQIPP (2015). Safety of long term proton pump inhibitors (PPIs).

[27] Sullivan S. D., Mauskopf J. A., Augustovski F.. (2014). Budget Impact Analysis—Principles of Good Practice: Report of the ISPOR 2012 Budget Impact Analysis Good Practice II Task Force. Value Health.

[28] Plehhova K., Haering M., Wray J., Coyle C., Melguizo-Ibáñez E., Kostev K. (2023). Prescribing patterns of proton pump inhibitors in Germany: a retrospective study including 472 146 patients. Pub Med.

[29] British National Formulary–Treatment summaries Proton pump inhibitors (BNF 90 September 2025–March 2026).

[30] Office for National Statistics (2024). Principal projection - England population in age groups.

[31] Office for National Statistics (2024). Principal projection: England population in age groups.

[32] (2023). Gaviscon Advance Aniseed Oral Suspension–Summary of Product Characteristics (SmPC) - (emc).

[33] (2022). Gaviscon Double Action Aniseed Oral Suspension–Summary of Product Characteristics (SmPC) - (emc).

[34] NHS Business Services Authority (2023). Prescription Cost Analysis – England – 2022-23.

[35] NHS England (2020). National Cost Collection for the NHS.

[36] Jones K. C., Weatherly H., Birch S.. (2022). Unit Costs of Health and Social Care 2022 Manual.

[37] (2022). Introduction to health technology evaluation: NICE health technology evaluations: the manual.

[38] Chau S. H., Sluiter R. L., Hugtenburg J. G., Wensing M., Kievit W., Teichert M. (2019). Correction to: Cost–utility and budget impact analysis for stopping the inappropriate use of proton pump inhibitors after cessation of NSAID or lowdose acetylsalicylic acid treatment. Drugs Aging.

[39] Murie J., Allen J., Simmonds R., de Wet C. (2012). Glad you brought it up: a patient-centred programme to reduce proton-pump inhibitor prescribing in general medical practice. Qual Prim Care.

[40] Cawston J., Evans N. (2007). Dyspepsia: key considerations for cost-effective management. Prescriber.

[41] NHS England (2022). Medicines optimisation.

[42] NHS England (2023). National medicines optimisation opportunities 2023/24.

[43] Connolly M., Bhatt A. (2008). PG17 AN ECONOMIC EVALUATION OF THE TWO LEADING ALGINTE THERAPIES IN THE UK: RESULTS FROM A LARGE LONGITUDINAL DATABASE. Value Health.

[44] Begg M., Tarhuni M., Fotso M. N.. (2023). Comparing the safety and efficacy of proton pump inhibitors and histamine-2 receptor antagonists in the management of patients with peptic ulcer disease: a systematic review. Cureus.

[45] NHS England Structured medication reviews and medicines optimisation.

[46] Anderson M., Francetic I. (2025). Adoption of clinical pharmacist roles in primary care: longitudinal evidence from English general practice. Br J Gen Pract.

