# Supplementary material for: Budget Impact Analysis of Stepping Down Patients from Long-Term Inappropriate Proton Pump Inhibitor Use to Episodic Alginate Treatment: NHS England Perspective
Source: J Health Econ Outcomes Res. 2025 Oct 30;12(2):163–72. doi: 10.36469/001c.144254 (PMC12579549; doi:10.36469/001c.144254)
Supplement: Online Supplementary Material [file jheor_2025_12_2_144254_308325.pdf]

Journal of Health Economics and Outcomes Research

Online Supplementary Material

Budget Impact Analysis of Stepping Down Patients from Long-Term Inappropriate Proton Pump Inhibitors Use to Episodic Alginate Treatment: NHS England Perspective. *JHEOR*. 2025;12(2):163-172. doi:10.36469/jheor.2025.144254

Figure S1: Stratification of Subgroups Based on Age and Duration of PPI Use

Table S1: Appropriate Indications for Long-Term PPI Use

Table S2: Drug Acquisition Cost, British National Formulary

Table S3: Adverse Effects by Class of Drug

Table S4: Distribution of Total Eligible Population Each Year by Treatment Agent Received

This supplementary material has been provided by the authors to give readers additional information about their work.

This is an open-access article distributed under the terms of the Creative Commons Attribution 4.0 International License (CCBY-4.0). View this license's legal deed at http://creativecommons.org/licenses/by/4.0 and legal code at http://creativecommons.org/licenses/by/4.0/legalcode for more information.

Figure S1. Stratification of Subgroups Based on Age and Duration of PPI Use

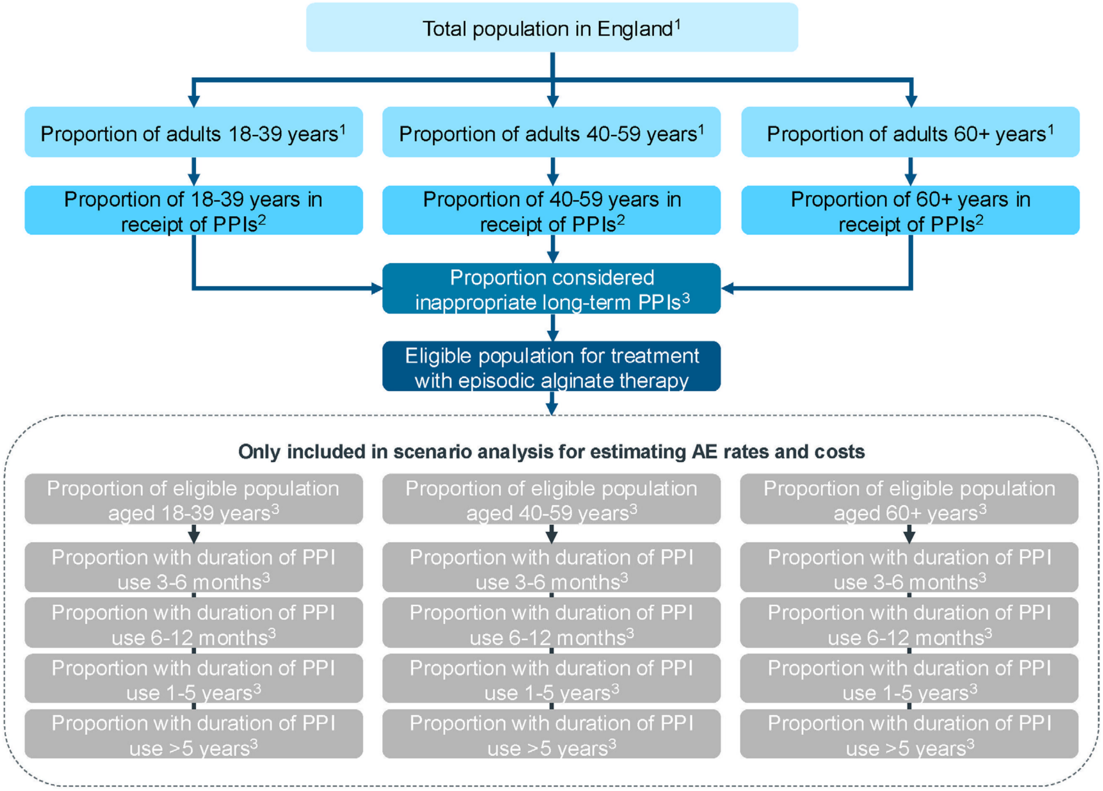

1. Office for National Statistics (ONS): 2021-based interim national population projections - Principal projection - England population in age groups (2023 values)
2. Abrahami et. al. 2020 Trends in acid suppressant drug prescriptions in primary care in the UK: a population-based cross-sectional study.
3. Plehhova et al. 2024 Prescribing practices for proton pump inhibitors among primary care physicians in England

Abbreviation: PPI, proton pump inhibitor.

Table S1. Appropriate Indications for Long-Term PPI Use[3,44]

Refractory or recurrent symptoms
Severe erosive esophagitis, refractory to initial treatment
Barrett's esophagus
Gastroprotection for long-term nonsteroidal anti-inflammatory use
Previous bleeding peptic ulcer disease with 2 failed attempts of *Helicobacter pylori* eradication
Zollinger-Ellison syndrome

Abbreviation: PPI, proton pump inhibitor.

Table S2. Drug Acquisition Cost, British National Formulary

Treatment	Administration Form	Bottle Size/ Tablet Strength	Pack Size (No. of Doses)	Cost per Pack, £[a]	Dose per Day, mg			Dose Equivalents Annually			Annual Drug Cost, £
					Low Dose	Full Dose	Double Dose	Low Dose	Full Dose	Double Dose	
Episodic alginate therapy	Liquid	500 ml	50	5.12	NA	NA	NA	NA	85	NA	8.70[b]
Esomeprazole	Tablet	20 mg	28	1.42	20	20	40	365	365	731	21.25
Lansoprazole	Tablet	30 mg	28	0.98	15	30	60	183	365	731	13.53
Omeprazole	Tablet	20 mg	28	0.75	10	20	40	183	365	731	10.35
Pantoprazole	Tablet	40 mg	28	0.93	20	40	80	183	365	731	12.84
Rabeprazole	Tablet	10 mg	28	3.56	10	20	40	365	731	1461	98.28

[a]Cost as presented in the British National Formulary in August 2024.
[b]As per Coyle et al,[25] during the 12-month period, an average of 1.7 bottles (500 ml each) of Gaviscon Advance were used per patient who stepped down and/or off proton pump inhibitors.

Table S3. Adverse Effects by Class of Drug

Parameter	No. of Adverse Effects					
	**Year 1**	**Year 2**	**Year 3**	**Year 4**	**Year 5**	**Total**
**Current scenario: Without episodic alginate therapy**						
Hip fracture	288	291	293	295	298	1465
Kidney disease	2435	2454	2474	2494	2514	12 371
CDI	2582	2603	2624	2645	2666	13 121
CAP	17 140	17 277	17 415	17 554	17 695	87 082
Total current scenario	22 446	22 625	22 806	22 989	23 173	114 038
**Projected scenario: With episodic alginate therapy**						
Patients successfully stepping down/off PPIs to episodic alginate treatment						
Hip fracture	21	20	24	28	32	124
Kidney disease	118	112	135	159	183	708
CDI	172	162	196	231	266	1027
CAP	1716	1623	1963	2308	2659	10 269
Total	2026	1917	2318	2726	3141	12 128
Patients not stepping down/off PPIs						
Hip fracture	260	263	260	257	253	1292
Kidney disease	2191	2224	2195	2166	2136	10 914
CDI	2324	2359	2329	2297	2266	11 575
CAP	15 426	15 656	15 454	15 248	15 038	76 823
Total	20 201	20 502	20 238	19 969	19 693	100 604
Total projected scenario	22 227	22 419	22 556	22 695	22 834	112 732
**Difference in adverse effects (Projected scenario – Current scenario): No. (%) of adverse effects**						
Hip fracture	−8 (−2.80)	−8 (−2.62)	−9 (−3.15)	−11 (−3.67)	−13 (−4.20)	−48 (−3.30)
Kidney disease	−125 (−5.14)	−118 (−4.83)	−143 (−5.79)	−169 (−6.76)	−194 (−7.72)	−750 (−6.06)
CDI	−87 (−3.35)	−82 (−3.15)	−99 (−3.78)	−117 (−4.40)	−134 (−5.03)	−518 (−3.95)
CAP	2 (0.01)	2 (0.01)	2 (0.01)	2 (0.01)	2 (0.01)	10 (0.01)
Total	−218 (−0.97)	−206 (−0.91)	−250 (−1.10)	−294 (−1.28)	−338 (−1.46)	−1307 (−1.15)

Abbreviations: CAP, community-acquired pneumonia; CDI, *Clostridium difficile* infection; PPI, proton pump inhibitors.

Table S4. Distribution of Total Eligible Population Each Year by Treatment Agent Received

Parameter	No. (%) of Patients					
	**Year 1**	**Year 2**	**Year 3**	**Year 4**	**Year 5**	**Total**
**Current scenario: Without episodic alginate therapy**						
Total eligible population (patients with long-term inappropriate use of PPIs)	7 727 893 (100)	7 789 703 (100)	7 852 007 (100)	7 914 809 (100)	7 978 114 (100)	39 262 527 (100)
	**Distribution of Total Eligible Population by Treatment Agent**					
Esomeprazole	277 753 (3.59)	279 975 (3.59)	282 214 (3.59)	284 471 (3.59)	286 747 (3.59)	1 411 161 (3.59)
Lansoprazole	3 482 472 (45.06)	3 510 326 (45.06)	3 538 402 (45.06)	3 566 703 (45.06)	3 595 231 (45.06)	17 693 134 (45.06)
Omeprazole	3 754 898 (48.59)	3 784 930 (48.59)	3 815 203 (48.59)	3 845 718 (48.59)	3 876 477 (48.59)	19 077 227 (48.59)
Pantoprazole	182 441 (2.36)	183 901 (2.36)	185 371 (2.36)	186 854 (2.36)	188 349 (2.36)	926 916 (2.36)
Rabeprazole	30 329 (0.39)	30 571 (0.39)	30 816 (0.39)	31 062 (0.39)	31 311 (0.39)	154 089 (0.39)
**Projected scenario: With episodic alginate therapy**						
Total eligible population (patients with long-term inappropriate use of PPIs)	7 727 893 (100)	7 789 703 (100)	7 852 007 (100)	7 914 809 (100)	7 978 114 (100)	39 262 527 (100)
	**Distribution of Total Eligible Population by Treatment Agent**					
Patients successfully stepping down/off PPIs to episodic alginate treatment[a]	772 789 (10.00)	781 041 (10.03)	935 035 (11.91)	1 091 442 (13.79)	1 250 292 (15.67)	4 830 600 (12.30)
Patients not stepping down/off PPIs						
Esomeprazole	249 978 (3.23)	251 903 (3.23)	248 608 (3.17)	245 243 (3.10)	241 809 (3.03)	1 237 541 (3.15)
Lansoprazole	3 134 225 (40.56)	3 158 360 (40.55)	3 117 041 (39.70)	3 074 859 (38.85)	3 031 803 (38.00)	15 516 288 (39.52)
Omeprazole	3 379 408 (43.73)	3 405 431 (43.72)	3 360 880 (42.80)	3 315 399 (41.89)	3 268 974 (40.97)	16 730 092 (42.61)
Pantoprazole	164 197 (2.12)	165 462 (2.12)	163 297 (2.08)	161 087 (2.04)	158 832 (1.99)	812 875 (2.07)
Rabeprazole	27 296 (0.35)	27 506 (0.35)	27 146 (0.35)	26 779 (0.34)	26 404 (0.33)	135 131 (0.34)

Abbreviation: PPI, proton pump inhibitor.

[a]For the projected scenario with alginate treatment, progressive increase in market share of alginate from 10% in year 1 to 20% in year 5 was assumed with a proportionate decrease in PPI market shares each year. However, the proportion of patients successfully stepping down/off PPIs to episodic alginate treatment considers patients with unsuccessful step down/off (ie, patients reverting back to their original PPIs after 12 months).
